# On the relation of psychiatric disorder and neural defect

**DOI:** 10.3389/fpsyg.2014.00040

**Published:** 2014-01-29

**Authors:** Jan-Hendrik Heinrichs

**Affiliations:** Institute for Neuroscience and Medicine 8: Ethics in the Neurosciences, Forschungszentrum Jülich GmbH in der Helmholtz GemeinschaftJülich, Germany

**Keywords:** psychiatric disorder, neural defect, functional explanation, mechanism, decomposition

The debate about the relation between psychiatric disorder and neural defect has produced different argumentative strategies for and against the identification of these two phenomena. I'll coin these strategies as (a) an ontological strategy, (b) an extensional strategy, and (c) an intensional strategy, on which I will focus in this article.

The first, ontological strategy takes the long road over a detailed characterization of the nature of psychiatric disorders and of neural defects. It then goes on to argue for a relation between these two ontological kinds. One anti-reductionist proposition of an ontological strategy can be found in Stier (2013). He provides a sketch of a theory of the nature of psychiatric diseases, claiming *inter alia* that psychiatric diseases are social constructions and intrinsically normative. He goes on to infer a non-reducibility thesis from these more or less ontological characterizations: “if the boundary between normality and mental disorder is a social construction such that the question of whether a certain kind of behavior is a disorder can only be judged against the background of this very convention, then the “disorderness” of a condition cannot be found on—and hence not be reduced to—the neuronal level” (Stier, 2013, p. 3). While Stier's argument fluctuates between an epistemic and ontological non-reducibility thesis, I take him to be talking about the nature of psychiatric diseases and thus about an ontological issue foremost.

The second, extensional strategy investigates the phenomena that fall into both categories in order to relate the categories. In principle one would have to identify all psychiatric diseases and their pathways in the brain. If one could identify at least one neural causal pathway for each psychiatric disease, all psychiatric diseases could be assumed to be brain diseases. Alternatively one could use a falsificatory strategy and look for one psychiatric disease, which does not have a neural causal pathway. Obviously both are only in principle and not practically viable options. Universally quantified statements and claims of non-existence are well-nigh impossible to prove. Typically, the extensional method is applied in exemplary research projects. Proponents of the identity or reducibility of psychiatric disease to neural phenomena try to show that a certain psychiatric disease can be explained with reference to neural phenomena. Opponents of reducibility try to identify psychiatric diseases for which an explanation via some neural, causal pathway is improbable. The extensional strategy has to combat severe methodological challenges as discussed by Kapur et al. (2012) cf. the discussion in Walter (2013): the power of neuro-psychiatric studies, the limited replication of studies, the reliance on extreme comparisons and of course, as Walter (2013) mentions, the ethical issues of research on the living brain.

The third, intensional strategy takes a slightly shorter road to elucidating the relation between psychiatric disorders and neural defects by discussing their explanatory roles: looking at the way the categories are defined and applied.

Turning to intensional methods requires some restriction in the use of the categories and concepts in question. Thus, instead of talking loosely about mental, psychiatric or psychological diseases or illnesses and physical, neural or brain based diseases or states the focus will be on psychiatric disorders and neural defects. The term “psychiatric disorder” will be used for two reasons: (1) “disorder” is the term used in the ICD 10 and in DSM IV and V; (2) I do not want to talk about the alleged mental/physical divide, but about the categorizations of the relevant scientific disciplines, in this case psychiatry; thus I do not use “mental disorder.” The term “neural defect” will be used for two similar reasons: (1) “defect” seems to be the weakest functional term (more on functional terms in a moment). (2) I use the word “neural” instead of “neuroscientific” because I want to refer to the broader category of defects detected by neurosciences as a whole, not just in single cell analyses (neuronal) or brain anatomy.

The contrast between the intensional strategy and the ontological and extensional strategies can be observed in Schramme (2013). While he does provide some details on the ontological positions in the debate of the mind-body problem in a quite extensive part of his article, his persuasive key arguments pertain to the explanatory roles of mental phenomena, especially psychiatric disorders, and require only a brief part of the text. Schrammes two convincing arguments for an irreducibility of psychiatric disorders to neural defects are (1) even membership in a neurological category will not explain, why the mental states realized by certain neural states are pathological. The ascription of pathology or of being disordered is dependent on the psychological level of explanation. (2) In an aside he observes that neurophysiological explanation does not even seek explanations of single event tokens.

According to Schramme's first argument, the identification of a neurological class of states all of which realize some psychiatric disorder would not suffice to explain the psychiatric disorder in question (Schramme, 2013, 5 f.). This argument can be further supported by some details on how the concepts in question are embedded in their explanatory projects.

“Psychiatric disorder” and “neural defect” are concepts from quite different disciplinary contexts, which slowly coalesce: “psychiatric disorder” is first and foremost a concept of a “-iatric” discipline, namely psychiatry. “Neural defect” is mainly a concept of neuroanatomy and -physiology. Both are functional concepts that serve a disciplinary purpose and have been shaped in order to do so. A concept is a functional concept by virtue of its embeddedness in functional explanation.

The functional explanatory strategy consists in decomposing a specific explanandum into a set of distinct parts and trying to show how the parts and their forces account for the original phenomenon: “[…] the analytical strategy proceeds by analyzing a disposition *d* of *a* into a number of other dispositions *d*_1_ … *d_n_* had by *a* or components of *a* such that programmed manifestations of the *d_i_* results in or amounts to a manifestation of *d*” (Cummins, 1975, p. 759).

The process of decomposition will be iterated during a functional explanation of complex systems, especially in explaining the behavior of an organism. The crucial question at each onset of decomposition is what to pick out as the phenomenon to explain. This decision recurs in every iteration of the decomposition procedure. In a decomposition of arm movement one has to decide whether to analyze the behavior of the muscles or that of the tendons or bones. On the next deeper level one chooses whether to analyze the behavior of cells or that of extracellular transport systems etc. Thus, just as the specific explanandum depends on the background theory, the explanatory path in functional explanation depends on the decompositional decisions.

Two distinct sub-types of this method give rise to the concepts of a psychiatric disorder and of a neural defect. Their differences are threefold:

1. Context of detection. Psychiatric disorders strike the observer as something to be explained and treated: Usually psychiatric disorders are abnormalities of behavioral patterns observed in terms of folk and scientific psychology. Either they themselves are the reason for an analysis of the behavioral pattern or the pattern has been the target of prior interest in psychology or cognitive science.
  Neural defects are further removed from casual attention: Neural defects are abnormalities in the working of causal pathways, in the parts and forces making up a phenomenon. They can only be found after an analysis. The abnormalities in these pathways can but need not result in abnormalities in behavior.
2. Context of action. The analysis of psychiatric disorders is driven by the desire to understand a behavioral abnormality and if possible to find a therapy or workaround. Neural defects in contrast are found in analysis driven by a purely explanatory research interest.
3. Last but not least, the context of explanation. Neural defects turn up when physiological phenomena are analyzed into physiological parts and their tempo-spatial relations. A neural defect primarily is defined within a mechanistic explanation of some neural phenomenon (Bechtel, 2009; Craver, 2013). Mechanistic explanation typically elucidates relatively complex behaviors of biological systems by the actions and interactions of their constituting subsystems. The actions and interactions of the subsystems in turn are explained by actions and interactions of their respective subsystems; insofar mechanistic explanation is a type of functional explanation as mentioned above (Craver, 2013). Mechanistic explanation is a special type of functional explanation however, because it strictly sticks to componential analysis, that is: the subsystems stand in a physical part-whole relation to the system, which gets explained. The main interest in ascribing a function and noticing a defect is explanatory. Something is a function because it contributes to some complex behavior in most homologs, which a scientist aims to explain. The word “function” could be replaced by “normal causal role.” Something is a defect, because in the more numerous homologs the causal pathways work differently. The word “defect” in this context could be replaced by “abnormality.”

A similar type of analysis can be found in cognitive science, with the not so minor variation that what gets explained are cognitive abilities and behavior, and they typically get analyzed into cognitive and affective sub-tasks and capacities. There are for example theories of long term memory, distinguishing it into subtasks of encoding and consolidation, storage and retrieval as well as into different subtypes like episodic, semantic, procedural, and priming memory. The explaining subsystem and the system, which get explained do not stand in a physical part-whole-relation. The decomposition is cybernetic and not componential.

While neural abnormalities can be considered defective only relative to explanatory interests, it seems to be possible to identify cognitive or affective defects beyond such an interest (contra Stier, 2013). If a person can't grasp objects in her right visual field, or can't remember words for more than a few moments, that seems to be a defect no matter what. This alleged obviousness of there being a defect stems from the close interdependence of cognitive science and psychiatry as regards their phenomena as well as their methodology. The psychiatric diagnosis of a defect and thus the psychiatric ascription of function and dysfunction is often prior to analysis in cognitive science. The discussion how to define functions in psychiatry is still on-going and vast [for an overview cf. Schramme (2010)]. None of the suggestions, however, takes recourse to analyzing complex behaviors into physical parts and forces as is done in mechanistic explanation. The explanantia of both methods, psychological and psychiatric, do not stand in a physical part-whole relation to the system and behavior explained.

To conclude: Neural defect and psychiatric disorder are defined within different types of analysis. One is componential, decomposing complex phenomena based on purely explanatory interests, ascribing function on the basis of comparison to homologs. The other is non-componential, decomposing complex phenomena based on interventionist interests, ascribing functions on the basis of systemic goals. The categories are thus neither identical nor bear an obvious intensional relation. As Schramme concludes, there is no reason for “discounting any of the two—neurophysiological or mental—perspectives. Mental illness is not reducible to brain illness, even when mental phenomena have their basis in the brain” (Schramme, 2013).

It is highly implausible that two categories based on different methods, research interests etc. are homomorphic, that is, can be related in a one to one style. As Schramme (2013, p. 6) points out as well, it would be more than surprising if for every taxonomic class of folk psychology, or, as I must add, of psychiatry there were one related type of neural defect or the other way around. As results in cognitive neuroscience, neuropsychiatry and related disciplines already show, the relation between neural defect and psychiatric disorder is much more complicated: different neural defects can result in the same disorder, sometimes a psychiatric disorder is caused by several coincidental neural defects etc. Thus, even if the metaphysical thesis, that all mental states are token identical to physical states is true, and I take it to be true, that does not help one bit in explaining or treating any of them.
